# Retraction: Heavy metal removal from MSS fly ash by thermal and chlorination treatments

**DOI:** 10.1038/srep40459

**Published:** 2017-01-16

**Authors:** Jingyong Liu, Jiacong Chen, Limao Huang

Scientific Reports 5: Article number: 1727010.1038/srep17270; published online: 11 25 2015; updated: 01 16 2017

The authors cannot guarantee the accuracy of the results and conclusions in this Article and wish to retract it.

The choice of chlorination reagents used in the experiments was inappropriate. The chlorinating agents used included NaCl, FeCl_3_•6H_2_O, MgCl_2_•6H_2_O, AlCl_3_ and CaCl_2_ but in order to accurately compare results, all the reagents used should have been hydrous or anhydrous chlorinating agents. During thermal treatment, hydrous chlorination reagents decompose and the crystallization water will become water vapour which may react with volatile metal chlorides and form higher stable oxides, which affects the distribution of heavy metals.

The treatment times used (3 h, 4 h, and 5 h) are too long and therefore not representative of real-world conditions used for the removal of heavy metals by incineration with fly ash.

The XRD analysis failed to take account of the complexity of the fly ash matrix, including minerals, heavy metals and other elements. The mineral phases are inaccurate and require further in-depth analysis.

Figure 6 is mislabeled. (a) “0% chlorination agent” should read “10% FeCl_3_”; (b) “10% CaCl_2_” should read “10% AlCl_3_”; (d) “10% NaCl” should read “10% CaCl_2_”; (e) “10% FeCl_3_” should read “10% NaCl”; (f) “10% AlCl_3_” should read “0% chlorination agent”.

All authors agree to the retraction of the Article.

